# Effect of anaesthesia modality on outcome in medium or distal vessel occlusion stroke: a post-hoc analysis of the DISTAL trial

**DOI:** 10.1093/esj/aakag088

**Published:** 2026-08-07

**Authors:** Silja Räty, Daniel Strbian, Laura Mannismäki, Mira Katan, Ronen R Leker, Robin Lemmens, Alejandro Tomasello, Julie Staals, Johannes Kaesmacher, Jan-Hendrik Buhk, Marta Olive Gadea, Juan F Arenillas, Nicolas Martinez-Majander, Steven D Hajdu, Patrik Michel, Volker Puetz, Flavio Bellante, Sylvie De Raedt, Daniel Bolliger, Vera Aebischer, Nikki Rommers, Alex Brehm, Marios Psychogios, Urs Fischer

**Affiliations:** Department of Neurology, Helsinki University Hospital and University of Helsinki, Helsinki, Finland; Department of Neurology, Helsinki University Hospital and University of Helsinki, Helsinki, Finland; Department of Neurology, Helsinki University Hospital and University of Helsinki, Helsinki, Finland; Department of Neurology, University Hospital Basel, University of Basel, Basel, Switzerland; Department of Neurology, Hadassah-Hebrew University Medical Center, Jerusalem, Israel; Department of Neurosciences, Experimental Neurology, KU Leuven, and Department of Neurology, University Hospitals Leuven, Leuven, Belgium; Interventional Neuroradiology Section, Vall d'Hebron University Hospital, Barcelona, Spain; Vall d'Hebron Research Institute, Vall d'Hebron Barcelona Hospital Campus, Barcelona, Spain; Department of Neurology, Cardiovascular research Institute Maastricht, Maastricht University Medical Center, Maastricht, the Netherlands; Department of Diagnostic and Interventional Neuroradiology, Inselspital, University Hospital Bern, University of Bern, Bern, Switzerland; Department of Neuroradiology, Asklepios Hospital St. Georg, Hamburg, Germany; Stroke Unit, Neurology Department, Vall d'Hebron University Hospital, Barcelona, Spain; Department of Neurology, Stroke Program and Clinical Neurosciences Research Group, Department of Medicine, University Hospital Valladolid, Valladolid, Spain; Department of Neurology, Helsinki University Hospital and University of Helsinki, Helsinki, Finland; Department of Diagnostic and Interventional Neuroradiology, Centre Hospitalier Universitaire Vaudois, Lausanne, Switzerland; Department of Neurology, Centre Hospitalier Universitaire Vaudois, Lausanne, Switzerland; Dresden Neurovascular Center, Faculty of Medicine and University Hospital Carl Gustav Carus, Technische Universität Dresden, Dresden, Germany; Department of Neurology, Faculty of Medicine and University Hospital Carl Gustav Carus, Technische Universität Dresden, Dresden, Germany; Department of Neurology, Hôpital Civil Marie Curie, Centre Hospitalier Universitaire Charleroi, Charleroi, Belgium; Department of Neurology, Universitair Ziekenhuis Brussel (UZ Brussel), NEUR Research Group, Vrije Universiteit Brussel (VUB), Brussels, Belgium; Clinic for Anaesthesia, Prehospital Emergency Medicine and Pain Therapy, University Hospital Basel, Basel, Switzerland; Department of Diagnostic and Interventional Neuroradiology, Radiology & Nuclear Medicine Clinic, University Hospital Basel, University of Basel, Basel, Switzerland; Department of Clinical Research, University of Basel, University Hospital Basel, Basel, Switzerland; Department of Diagnostic and Interventional Neuroradiology, Radiology & Nuclear Medicine Clinic, University Hospital Basel, University of Basel, Basel, Switzerland; Department of Diagnostic and Interventional Neuroradiology, Radiology & Nuclear Medicine Clinic, University Hospital Basel, University of Basel, Basel, Switzerland; Department of Neurology, University Hospital Bern, University of Bern, Bern, Switzerland

**Keywords:** anaesthesia, endovascular treatment, medium or distal vessel occlusion, stroke

## Abstract

**Introduction:**

Of the 3 published trials, only 1 showed possible efficacy of endovascular treatment (EVT) compared to best medical treatment (BMT) alone for medium or distal vessel occlusions (MDVO), whereas the others were neutral. Procedural conditions may affect the outcome.

**Patients and methods:**

This is a post-hoc analysis of the randomised, controlled DISTAL trial, conducted in 55 centers between 12/2021 and 7/2024. Patients with isolated MDVO within 24 hours of last seen well randomised to EVT plus BMT were treated under general anaesthesia (GA), conscious sedation (CS) or local anaesthesia (LA) at investigators’ discretion. Endovascular treatment under different anaesthesia modalities was compared head-to-head, and to BMT alone.

**Results:**

Among 491 patients included in the analysis, 224 received EVT plus BMT (108 GA, 64 LA, 52 CS) and 267 received BMT alone. Compared to BMT alone, EVT under CS was associated with worse 90-day modified Rankin Scale (median 2.5 vs 2.0; adjusted odds ratio [aOR] 0.51 [95% CI 0.29–0.90]), whereas no difference was observed between EVT under other anaesthesia modalities and BMT alone. Symptomatic intracranial haemorrhage occurred more frequently after EVT under GA compared to BMT alone (8.3% vs 2.6%; aOR 3.37 [95% CI, 1.22–9.66]). LA was associated with higher odds of better functional outcome (aOR 2.11 [95% CI, 1.09–4.12]) and lower mortality (aOR 0.28 [95% CI, 0.08–0.95]) compared to CS, whereas there was no difference to GA.

**Discussion and conclusion:**

The lack of superiority of EVT plus BMT over BMT alone in DISTAL may not have been driven by anaesthesia modality.

## Graphical Abstract

**Graphical Abstract:**
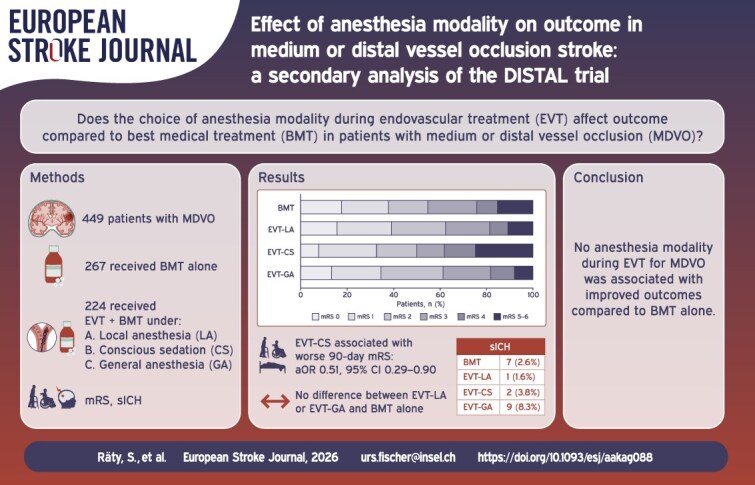

## Introduction

Endovascular treatment (EVT) has revolutionised the treatment of acute ischaemic stroke, demonstrating significant benefits in patients with large vessel occlusion (LVO).^1^ However, regarding medium or distal vessel occlusions (MDVO), only 1 randomised controlled trial (RCT) showed possible efficacy of EVT compared to best medical treatment (BMT) in patients with higher severity of symptoms,^2^ whereas 2 other RCTs were neutral.^3^^,^^4^ Possible explanations for lack of benefit of EVT in MDVO include a lower recanalization rate and increased risk of complications when targeting smaller vessels. Therefore, optimising procedural conditions and patient stability through appropriate sedation may be even more relevant than in LVO; yet, approaches to anaesthesia for MDVO-EVT vary widely.^5^

Several trials have investigated the impact of anaesthesia modalities on outcome after EVT for LVO.^6–13^ Their meta-analyses showed that patients treated under general anaesthesia (GA) achieve recanalization more often than patients receiving non-GA but have a higher risk of hemodynamic instability and post-procedural pneumonia.^14^^,^^15^ However, the impact of sedation modality on functional outcome remains elusive. Regarding non-GA approaches, a meta-analysis on studies comparing local anaesthesia (LA) and conscious sedation (CS) found no differences in outcomes.^16^

## Aims and hypothesis

In the Endovascular Therapy plus Best Medical Treatment (BMT) versus BMT Alone for Medium Vessel Occlusion Stroke (DISTAL) trial, no difference in functional outcomes was observed between EVT plus BMT versus BMT alone in patients with MDVO.^3^ In the EVT plus BMT group, the patients were treated either under GA, LA or CS. We hypothesised that patient movement under LA or CS could impair recanalization and safety and thus influence the efficacy of EVT. Hence, the aim of the study was to evaluate outcomes after EVT plus BMT under different anaesthesia modalities compared to BMT alone in patients with MDVO. Additionally, we aimed to compare outcomes and procedural metrics among EVT plus BMT patients treated under GA, LA or CS.

## Methods

This is a post-hoc secondary as-treated analysis of the DISTAL trial, an investigator-initiated, assessor-blinded, RCT conducted in 55 centres in 11 countries between December 2021 and July 2024. The trial investigated whether EVT plus BMT is superior to BMT alone in reducing disability and mortality in patients with acute ischemic stroke due to isolated MDVO. The protocol and primary results of the trial have been published.^3^^,^^17^

The main eligibility criteria included age ≥18 years, not residing in a nursing facility or being bedridden, acute ischemic stroke due to isolated MDVO, a National Institutes of Health Stroke Scale (NIHSS) score ≥4 (or lower in disabling symptoms), and treatment possible within 24 hours of last seen well. MDVO was defined as occlusion of the non-dominant or co-dominant M2, M3 or M4 segment of the middle cerebral artery; the A1, A2 or A3 segment of the anterior cerebral artery; or the P1, P2 or P3 segment of the posterior cerebral artery. Patients were randomised to receive EVT plus BMT or BMT alone.

In this post-hoc analysis, patients were divided into groups based on anaesthesia type during EVT: (1) EVT-GA, (2) EVT-LA and (3) EVT-CS, and each group was compared with BMT alone. Anaesthesia modality was independently selected by the treating physician in each centre and administered following local protocols. Information on anaesthesia modality was available for all patients, and only patients undergoing EVT received anaesthesia. This analysis included all DISTAL patients, except for those who did not undergo EVT due to recanalization or inaccessible occlusion, crossed over between EVT plus BMT and BMT alone, had anaesthesia converted from CS/LA to GA, or had missing data on the primary outcome. Because there were no data on conversions between LA and CS, these patients were included in the analysis.

The primary outcome of the study was the 90-day modified Rankin Scale (mRS) score, with categories 5 and 6 collapsed, assessed by a blinded evaluator during a follow-up visit or structured telephone interview. Secondary outcomes included mRS 0–2, mRS 0–1, mortality within 90 days, symptomatic intracranial haemorrhage (sICH) within 24 hours according to the modified Safe Implementation of Thrombolysis in Stroke–Monitoring Study criteria, and the proportion of patients with any serious adverse event (SAE).^18^ The rate of successful reperfusion was defined as a modified Thrombolysis in Cerebral Infarction (mTICI) score of 2b–3. Imaging data were evaluated centrally at an independent core laboratory.

### Statistical analyses

The primary outcome of mRS shift towards lower (ie, better) scores was analysed using unadjusted and adjusted mixed-effects ordinal regression models after confirming the proportional odds assumption. A random intercept was added to acknowledge clustering of patients within study centres, and predefined confounders (age, pre-stroke mRS, NIHSS at admission, IVT, occlusion location and time since last seen well [≤6 hours vs 6–24 hours]) were included as fixed effects. The predictor of interest had 4 levels: BMT alone (reference level), EVT-GA, EVT-LA and EVT-CS. Results are presented as (common) odds ratios, with 95% confidence intervals (CI).

Secondary binary outcomes were analysed using a logistic regression model. Due to convergence issues related to the large number of centres, the random intercept was removed from the logistic regression models; however, it did not show a strong effect on the results in executable analyses. Analyses on safety outcomes were adjusted for NIHSS at admission. Confounder selection followed the primary DISTAL analyses.

We replicated the primary analyses within the EVT group for unadjusted head-to-head (pairwise) comparisons between anaesthesia modalities. For these comparisons, successful reperfusion was added as an outcome, adjusting for age, NIHSS at admission, IVT, occlusion location and time since last seen well. Additionally, 5 supplementary sensitivity analyses were performed to assess the influence of crossover patients and potential overlap between anaesthesia modalities. First, patients who crossed over from BMT alone to EVT plus BMT were assigned to an EVT group based on anaesthesia modality, and those who did not undergo EVT were included in the BMT alone group irrespective of whether they had received some sedation. Second, patients crossing over to BMT alone were allocated to the group only if they received no anaesthesia, while patients who had GA, LA or CS were assigned to the respective EVT groups, irrespective of whether EVT was performed. Third, a non-GA group (LA + CS) was compared to GA and to BMT alone to account for possible crossover between LA and CS. Finally, modalities using any sedatives (GA and CS) were merged and compared to LA.

No adjustments for multiple comparisons were made, and missing data were excluded. All analyses were performed with R software (version 4.4.1).

## Results

**Figure 1:**
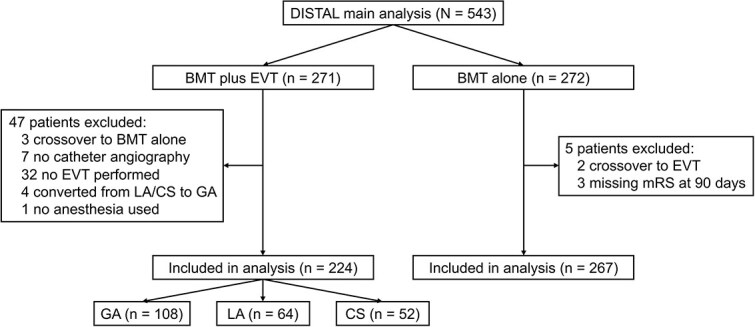
Flow diagram of the study. Note: BMT = best medical treatment; EVT = endovascular treatment; GA = general anaesthesia; LA = local anaesthesia; CS = conscious sedation; mRS = modified Rankin scale.

The final sample comprised 491 patients (267 in BMT, 108 in EVT-GA, 64 in EVT-LA and 52 in EVT-CS) after excluding 52 patients (Figure 1). There were no major differences in age, pre-stroke mRS, baseline NIHSS score or stroke aetiology (Table 1).

**Table 1:** Baseline characteristics and procedural metrics of the treatment groups.

	BMT (*n* = 267)	EVT-GA (*n* = 108)	EVT-LA (*n* = 64)	EVT-CS (*n* = 52)
**Age (y), median (IQR)**	78.0 (68.0–84.0)	75.0 (67.8–82.0)	77.0 (66.8–85.0)	80.0 (68.8–84.2)
**Age >80 y, *n* (%)**	110 (41.2)	34 (31.5)	25 (39.1)	25 (48.1)
**Sex male, *n* (%)**	145 (54.3)	66 (61.1)	30 (46.9)	35 (67.3)
**Pre-stroke mRS, *n* (%)**				
** 0–1**	210 (78.9)	91 (84.3)	51 (79.7)	42 (80.8)
** 2**	31 (11.7)	5 (4.6)	9 (14.1)	7 (13.5)
** 3–4**	25 (9.4)	12 (11.1)	4 (6.2)	3 (5.8)
**Hypertension, *n* (%)**	174 (65.2)	73 (67.6)	37 (57.8)	38 (73.1)
**Hypercholesterolemia, *n* (%)**	122 (45.7)	47 (43.5)	28 (43.8)	20 (38.5)
**Diabetes mellitus, *n* (%)**	65 (24.3)	22 (20.4)	13 (20.3)	15 (29.4)
**Previous stroke or TIA, *n* (%)**	59 (22.1)	22 (20.4)	19 (29.7)	12 (23.1)
**Atrial fibrillation, *n* (%)**	67 (25.1)	27 (25.0)	17 (26.6)	12 (23.1)
**Heart disease, *n* (%)**	80 (30.0)	29 (26.9)	19 (29.7)	10 (19.2)
**NIHSS on admission, median (IQR)**	6.0 (5.0–9.0)	6.0 (5.0–9.0)	6.0 (4.0–8.0)	5.0 (4.8–8.2)
**Occlusion site, *n* (%)**				
** A1**	1 (0.4)	0	0	0
** A2**	5 (1.9)	2 (1.9)	1 (1.6)	4 (7.7)
** A3**	5 (1.9)	5 (4.6)	2 (3.1)	1 (1.9)
** M1**	0	0	1 (1.6)	0
** M2**	108 (40.4)	60 (55.6)	33 (51.6)	20 (38.5)
** M3**	81 (30.3)	18 (16.7)	13 (20.3)	15 (28.8)
** M4**	0	1 (0.9)	1 (1.6)	1 (1.9)
** No occlusion**	2 (0.7)	0	0	0
** P1**	13 (4.9)	6 (5.6)	3 (4.7)	4 (7.7)
** P2**	41 (15.4)	15 (13.9)	6 (9.4)	6 (11.5)
** P3**	11 (4.1)	1 (0.9)	4 (6.2)	1 (1.9)
**Right-sided occlusion, *n* (%)**	109 (41.1)	57 (52.8)	19 (29.7)	26 (50.0)
**Systolic BP (mmHg), mean (SD)**	153.9 (23.3)	151.9 (24.9)	155.2 (29.2)	151.8 (26.8)
**Plasma glucose (mmol/l), median (IQR)**	7.3 (6.0–9.7)	6.8 (5.9–9.7)	6.8 (6.0–8.6)	6.6 (5.6–8.0)
**Stroke aetiology, *n* (%)**				
** Large-artery atherosclerosis**	29 (10.9)	13 (12.0)	7 (10.9)	6 (11.8)
** Cardioembolism**	130 (48.9)	53 (49.1)	32 (50.0)	24 (47.1)
** Other determined aetiology**	8 (3.0)	1 (0.9)	2 (3.1)	3 (5.9)
** More than one cause**	7 (2.6)	4 (3.7)	0	2 (3.9)
** Unknown**	92 (34.6)	37 (34.3)	23 (35.9)	16 (31.4)
**IVT, *n* (%)**	184 (68.9)	73 (67.6)	34 (53.1)	29 (55.8)
**Absence of demarcation, *n* (%)**	245 (93.2)	103 (98.1)	57 (90.5)	50 (96.2)
**Tandem occlusion, *n* (%)**	19 (7.3)	6 (5.6)	2 (3.1)	2 (3.8)
**Time LSW to imaging (h), median (IQR)**	3.5 (1.6–9.7)	3.4 (1.5–9.3)	5.5 (2.0–11.0)	3.0 (1.7–6.9)
**Time LSW to randomisation (h), median (IQR)**	3.9 (2.3–10.0)	3.9 (2.2–9.1)	5.7 (2.5–11.6)	3.9 (2.5–7.2)
**Time LSW to randomisation, *n* (%)**				
** ≤4.5 h**	147 (55.9)	61 (57.0)	28 (43.8)	29 (55.8)
** 4.5-6 h**	21 (8.0)	11 (10.3)	4 (6.2)	7 (13.5)
** 6-12 hours**	47 (17.9)	16 (15.0)	20 (31.2)	10 (19.2)
** 12-24 h**	48 (18.3)	19 (17.8)	12 (18.8)	6 (11.5)
**Time LSW to puncture (h), median (IQR)**		4.7 (2.9–11.4)	6.5 (3.2–12.3)	4.7 (3.0–9.0)
**Time imaging to puncture (min), median (IQR)**		72 (57.8–91.5)	64.0 (50.5–83.5)	77.0 (53.8–96.2)
**Time randomisation to puncture (min), median (IQR)**		39.4 (30.1–48.1)	29.7 (20.5–58.5)	33.2 (20.8–47.1)
**Time randomisation to reperfusion (min), median (IQR)**		79.6 (66.8–106.1)	66.7 (44.6–107.2)	77.4 (53.3–86.8)
**First-line EVT strategy, *n* (%)**				
** 1. Stent retriever only**		17 (15.7)	10 (15.6)	7 (13.5)
** 2. Aspiration only**		17 (15.7)	12 (18.8)	7 (13.5)
** 3. Combined approach**		70 (64.8)	40 (62.5)	36 (69.2)
** 4. Intra-arterial thrombolysis**		4 (3.7)	2 (3.1)	1 (1.9)
** 5. Stenting or PTA**		0	0	1 (1.9)
**Number of EVT passes, median (IQR)**		1.0 (1.0–2.0)	1.0 (1.0–2.0)	2.0 (1.0–3.0)
**Balloon guide catheter use, *n* (%)**		42 (38.9)	32 (50.0)	10 (19.2)
**Successful reperfusion (mTICI 2b-3), *n* (%)**		82 (78.1)	53 (82.8)	39 (78.0)
**Near complete perfusion (mTICI 2c-3), *n* (%)**		61 (58.1)	42 (65.6)	31 (62.0)
**Vessel perforation, *n* (%)**		6 (5.6)	0	0
**Access hematoma, *n* (%)**		1 (0.9)	5 (7.8)	2 (3.8)
**Access haemorrhage, *n* (%)**		1 (0.9)	0	0

Abbreviations: BMT = best medical treatment; BP = blood pressure; CS = conscious sedation; EVT = endovascular treatment; GA = general anaesthesia; IVT = intravenous thrombolysis; LA = local anaesthesia; LSW = last seen well; mRS = modified Rankin Scale; mTICI = modified Thrombolysis in Cerebral Infarction; NIHSS = National Institutes of Health Stroke Scale; PTA = percutaneous transluminal angioplasty.

Successful reperfusion was achieved in 78.1% of EVT-GA cases, 82.8% in EVT-LA cases and 78.0% in EVT-CS cases (Table 1). Six vessel perforations occurred in the EVT-GA group compared to none in the other 2 groups.

**Figure 2:**
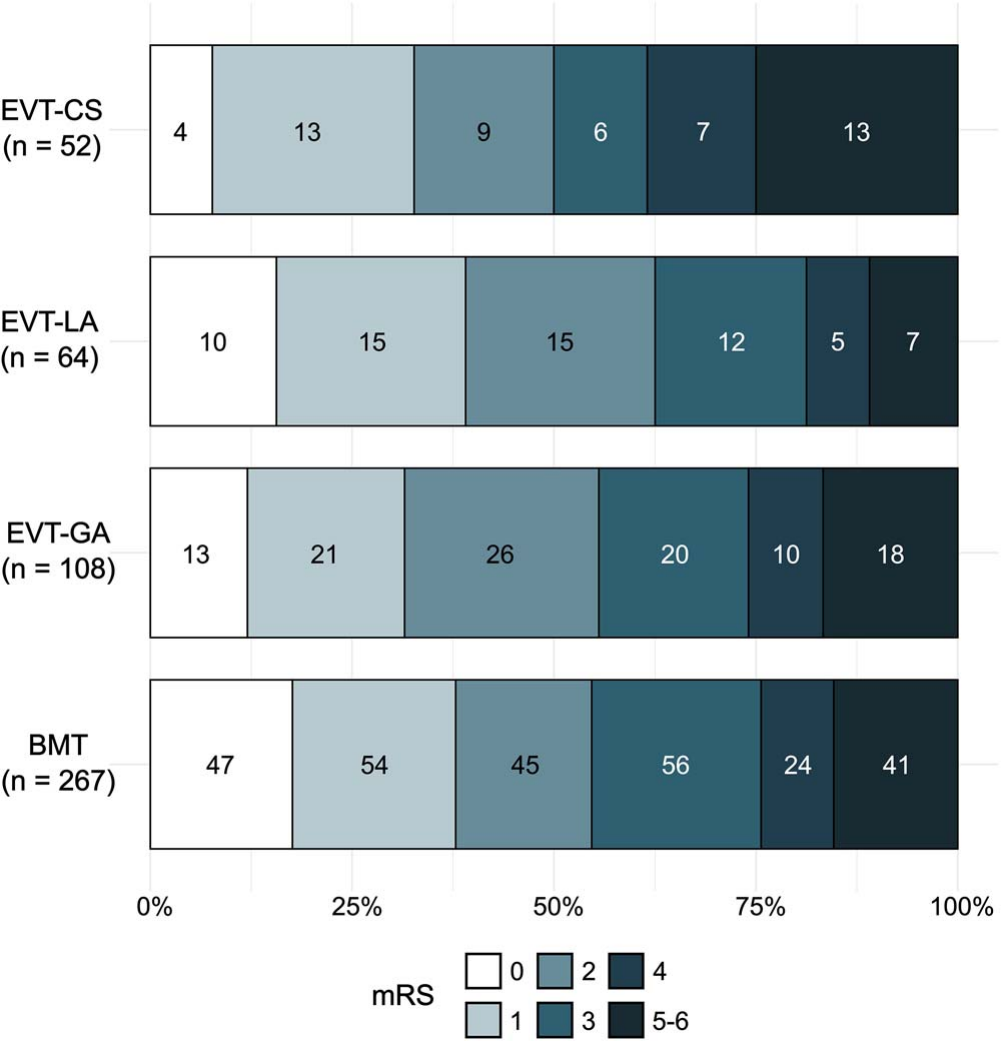
Distribution of modified Rankin scale at 90 days in the treatment groups. Note: EVT = endovascular treatment; CS = conscious sedation; LA = local anaesthesia; GA = general anaesthesia; BMT = best medical treatment; mRS = modified Rankin scale.

The distribution of mRS scores at 90 days is illustrated in Figure 2. EVT-CS was associated with lower odds of a shift towards lower mRS scores compared to BMT alone (adjusted odds ratio [aOR] 0.51, 95% CI, 0.29–0.90), whereas EVT-GA and EVT-LA did not differ from BMT alone (Table 2). Other efficacy outcomes were similar between the groups. EVT-GA was associated with higher odds for sICH compared to BMT alone (aOR 3.37, 95% CI, 1.22–9.66), but there were no differences in mortality or SAE occurrence. At least 1 SAE was reported in 27.3% (BMT), 33.3% (EVT-GA), 26.6% (EVT-LA) and 34.6% (EVT-CS) of the patients, and aspiration pneumonia in 1.9%, 3.7%, 4.7% and 1.9%, respectively.

**Table 2:** Outcomes according to anaesthesia modality during endovascular treatment and comparison to best medical treatment.

	BMT (*n* = 267)	EVT-GA (*n* = 108)	EVT-LA (*n* = 64)	EVT-CS (*n* = 52)	EVT-GA vs BMT		EVT-LA vs BMT		EVT-CS vs BMT	
					OR (95% CI)	aOR (95% CI)	OR (95% CI)	aOR (95% CI)	OR (95% CI)	aOR (95% CI)
**mRS at 90 d, median (IQR)^a^**	2.0 (1.0–3.0)	2.0 (1.0–4.0)	2.0 (1.0–3.0)	2.5 (1.0–4.2)	0.89 (0.59–1.33)^b^	0.78 (0.51–1.19)^b^	1.15 (0.71–1.87)^b^	1.21 (0.73–2.01)^b^	0.58 (0.33–1.02)^b^	0.51 (0.29–0.90)^b^
**mRS 0–2 at 90 d, *n* (%)^a^**	146 (54.7)	60 (55.6)	40 (62.5)	26 (50.0)	1.04 (0.66–1.63)	0.86 (0.50–1.51)	1.38 (0.79–2.45)	1.45 (0.72–2.97)	0.83 (0.46–1.51)	0.77 (0.38–1.56)
**mRS 0–1 at 90 d, *n* (%)^a^**	101 (37.8)	34 (31.5)	25 (39.1)	17 (32.7)	0.76 (0.47–1.21)	0.66 (0.39–1.11)	1.05 (0.60–1.84)	1.00 (0.54–1.82)	0.80 (0.42–1.48)	0.76 (0.38–1.49)
**Death within 90 d, *n* (%)^c^**	29 (10.9)	13 (12.0)	4 (6.2)	10 (19.2)	1.12 (0.54–2.21)	1.16 (0.56–2.30)	0.55 (0.16–1.46)	0.59 (0.17–1.58)	1.95 (0.85–4.20)	2.12 (0.91–4.60)
**sICH, *n* (%)^c^**	7 (2.6)	9 (8.3)	1 (1.6)	2 (3.8)	3.38 (1.23–9.68)	3.37 (1.22–9.66)	0.59 (0.03–3.40)	0.57 (0.03–3.29)	1.49 (0.22–6.36)	1.45 (0.21–6.23)
**Any SAE, *n* (%)^c^**	73 (27.3)	36 (33.3)	17 (26.6)	18 (34.6)	1.33 (0.82–2.15)	1.35 (0.83–2.19)	0.96 (0.51–1.75)	1.01 (0.53–1.85)	1.41 (0.74–2.62)	1.47 (0.76–2.75)

^a^Adjusted for age, pre-stroke mRS, National Institutes of Health Stroke Scale (NIHSS) at admission, intravenous thrombolysis, occlusion location, and time since last seen well (≤6 hours vs 6–24 hours). ^b^Common OR. ^c^Adjusted for NIHSS at admission. Abbreviations: (a)OR = (adjusted) odds ratio; CI = confidence interval; BMT = best medical treatment; CS = conscious sedation; EVT = endovascular treatment; GA = general anaesthesia; LA = local anaesthesia; mRS = modified Rankin Scale; sICH = symptomatic intracranial haemorrhage.

In the subgroup analysis comparing anaesthesia modalities among EVT-treated patients, LA was associated with higher odds of lower mRS scores (aOR 2.11, 95% CI, 1.09–4.12) and lower mortality (aOR 0.28, 95% CI, 0.08–0.95) compared to CS, while no other between-group differences were observed (Table 3). In 2 sensitivity analyses including the crossover patients, results were consistent with the main analyses. Finally, merging LA and CS into a non-GA group did not affect the primary outcome either compared to BMT alone or to EVT-GA, nor merging GA and CS into an any-sedatives group, compared to EVT-LA (Tables S1 and S2).

**Table 3:** Outcome comparison between different anaesthesia modalities during endovascular treatment.

	EVT-LA vs EVT-CS		EVT-LA vs EVT-GA		EVT-CS vs EVT-GA	
	OR (95% CI)	aOR (95% CI)	OR (95% CI)	aOR (95% CI)	OR (95% CI)	aOR (95% CI)
**mRS at 90 d^a^**	1.87 (0.96–3.66)^b^	2.11 (1.09–4.12)^b^	1.41 (0.80–2.47)^b^	1.57 (0.89–2.75)^b^	0.75 (0.41–1.39)^b^	0.74 (0.40–1.37)^b^
**mRS 0–2 at 90 d^a^**	1.67 (0.79–3.50)	1.85 (0.77–4.42)	1.33 (0.71–2.51)	1.74 (0.81–3.72)	0.80 (0.41–1.55)	0.94 (0.44–2.03)
**mRS 0–1 at 90 d^a^**	1.32 (0.61–2.84)	1.28 (0.55–2.98)	1.40 (0.73–2.66)	1.57 (0.77–3.18)	1.06 (0.52–2.15)	1.23 (0.56–2.70)
**Death within 90 d^c^**	0.28 (0.08–0.95)	0.28 (0.08–0.95)	0.49 (0.15–1.56)	0.49 (0.15–1.57)	1.74 (0.71–4.28)	1.74 (0.71–4.29)
**sICH^c^**	0.40 (0.04–4.50)	0.39 (0.04–4.47)	0.18 (0.02–1.41)	0.17 (0.02–1.39)	0.44 (0.09–2.11)	0.44 (0.09–2.09)
**Any SAE^c^**	0.68 (0.31–1.52)	0.68 (0.31–1.51)	0.72 (0.37–1.43)	0.72 (0.36–1.42)	1.06 (0.53–2.13)	1.05 (0.52–2.12)
**Successful reperfusion^d^**	1.36 (0.53–3.45)	1.25 (0.48–3.28)	1.35 (0.62–3.09)	1.41 (0.63–3.31)	0.99 (0.45–2.31)	1.12 (0.49–2.72)

^a^Adjusted for age, pre-stroke mRS, National Institutes of Health Stroke Scale (NIHSS) at admission, intravenous thrombolysis (IVT), occlusion location, and time since last seen well (≤6 hours vs 6–24 hours). ^b^Common OR. ^c^Adjusted for NIHSS at admission. ^d^Adjusted for age, NIHSS at admission, IVT, occlusion location, and time since last seen well. Abbreviations: (a)OR = (adjusted) odds ratio; CS = conscious sedation; CI = confidence interval; EVT = endovascular treatment; GA = general anaesthesia; LA = local anaesthesia; mRS = modified Rankin Scale; sICH = symptomatic intracranial haemorrhage.

## Discussion

In this post-hoc analysis of the DISTAL trial, EVT plus BMT was not associated with improved 90-day functional outcome compared to BMT alone regardless of anaesthesia modality. Instead, EVT-CS was associated with worse functional outcomes compared to BMT alone, and the odds of sICH were higher in the EVT-GA group.

For anaesthesia during EVT for MDVO, available data are mainly observational. Individual studies have reported more frequent successful reperfusion both after CS compared to GA,^19^ and after GA compared to non-GA,^20^^,^^21^ a higher sICH rate after GA compared to CS,^22^ or no differences in functional or safety outcomes between GA and non-GA.^20^^,^^21^^,^^23–25^ However, a meta-analysis on observational studies favoured non-GA over GA for 90-day mRS 0–1 and reduced mortality.^20^

In this study, EVT-CS was associated with worse functional outcomes compared to both BMT and EVT-LA, as well as higher mortality compared to EVT-LA. CS is often defined as the use of any systemic sedative medication to prevent pain, anxiety or movement without invasive ventilation support, whereas LA involves solely application of local anaesthetics at the puncture site. Therefore, CS may range from continuous infusions to intermittent small doses of sedatives. This variability complicates comparisons between CS and other anaesthesia modalities, as the risk of hemodynamic instability or aspiration during CS may differ considerably based on the approach. In addition to the procedure characteristics inherent to a conversion from LA to CS, such as patient movement, the patients who were treated under CS were older, which may also have affected the outcomes. Nevertheless, a meta-analysis of observational studies comparing CS and LA found no difference in outcomes between the 2 modalities, despite variable results of individual studies.^16^

Overall, LA was more frequently associated with functional independence compared to other anaesthesia modalities, even though the results of the supplementary analysis should be interpreted cautiously due to limited statistical power. Patients treated under LA had shorter randomisation-to-reperfusion times, similar rates of successful reperfusion, and few procedural complications despite a potentially higher risk of patient movement during EVT. Notably, overall baseline symptom severity did not differ between the groups.

In contrast to most previous studies, GA did not improve reperfusion but was associated with an increased risk of sICH. Higher reperfusion rates have mainly been detected in studies on LVO,^14^^,^^15^ whereas results on MDVO have been less conclusive.^20^ The more controlled procedural conditions during GA might increase confidence to target smaller vessels but not be sufficient to overcome the risk of vessel perforation related to anatomical challenges. Moreover, neurological deterioration during EVT is less likely to be observed under GA. We lack data on hemodynamic instability during EVT, but previous studies have mainly reported GA-related hypotension,^14^ which is unlikely a major contributor to sICH. In addition, all vessel perforations and most sICHs occurred in the GA group. This suggests that operators may have selected GA for more technically demanding procedures involving tortuous anatomy, more distal targets or greater procedural complexity. Therefore, the observed association between GA and poorer safety outcomes may partly reflect the complexity of the procedure rather than the anaesthesia modality itself.

In the DISTAL trial, GA was used in 49.6% of patients randomised to EVT, which is in contrast to the HERMES collaboration that reported a GA rate of 30% among patients with anterior circulation LVO.^26^ As patients enrolled in LVO trials had more severe symptoms than those in MDVO trials, this difference likely reflects centre- or interventionalist-related preferences rather than solely patient-specific factors.^5^

### Limitations

The results of this post-hoc analysis should be interpreted cautiously, according to the principles of non-randomised studies. This entails the risk of selection, as well as confounding and conversion bias. The choice of anaesthesia modality may have been influenced by the patient’s condition, which could have affected the outcome and biased the comparison either against GA or in favour of LA or CS. Factors potentially affecting the choice of anaesthesia were considered in the adjusted analysis and by adding a random intercept, but data on patient agitation or instability were unavailable, so residual confounding may remain. Additionally, since the initially planned anaesthesia modality was not collected, the current analysis should be described as a post-hoc, as-treated analysis. Although the known LA/CS-to-GA conversion patients were excluded, we cannot fully account for or quantify this bias. We performed sensitivity analyses using the available recorded anaesthesia information, but they can only show whether the observed results were robust under alternative classifications. Second, anaesthesia modality was reported by participating centres without central adjudication, and further data on medications, sedation depth, airway management strategies or monitoring practices and procedural metrics under different modalities were not collected. This means that description allowing replication of these interventions is not possible. Similarly, the physiological metrics, such as partial pressure of arterial carbon dioxide and blood pressure of patients, may have been affected by different anaesthetics, and the vasodilating or vasoconstricting effects of different anaesthesia agents could have acted as potential confounders. While misclassification between GA and non-GA is unlikely, uncertainty between CS and LA cannot be excluded. Third, this post-hoc analysis was not powered for comparisons between non-randomised anaesthesia modalities. The 3-way split resulted in small groups, particularly for LA and CS, and therefore clinically relevant differences cannot be excluded. The width of the CIs should be interpreted as reflecting substantial uncertainty. Finally, as the BMT alone group did not receive anaesthesia, it was not possible to conduct treatment interaction analyses based on anaesthesia modality.

In conclusion, according to this post-hoc analysis, the lack of superiority of EVT plus BMT over BMT alone in DISTAL may not have been driven by anaesthesia modality, as none of the modalities within the EVT group was associated with improved outcomes.

## Supplementary Material

Supplemental_material_revision_aakag088

## Data Availability

Metadata describing the type, size and content of the DISTAL trial dataset will be published along with the study protocol on the open repository Zenodo (https://zenodo.org/). Researchers wishing to access the full dataset can file a request with the Data Access Committee of the Medical Faculty of the University of Basel (MF-DAC—med-dac@unibas.ch). The MF-DAC will act as an independent assessor of the request and grant access to the dataset if all ethical, legal and scientific conditions are met. Data will be available 3 years after the publication of the main trial results.
