# Supplementary material for: Effect of anaesthesia modality on outcome in medium or distal vessel occlusion stroke: a post-hoc analysis of the DISTAL trial
Source: Eur Stroke J. 2026 Aug 7;11(8):aakag088. doi: 10.1093/esj/aakag088 (PMC13450685; doi:10.1093/esj/aakag088)
Supplement: Supplementary file 1 [file supplemental_material_revision_aakag088.docx]

**Supplemental material**

**Table S1.** Unadjusted sensitivity analyses on outcome comparison between different anesthesia modalities and best medical treatment

|  | **mRS at 90 d** | **mRS 0-2 at 90 d** |
| --- | --- | --- |
| **Sensitivity analysis 1** |  |  |
| EVT-GA vs. BMT  OR (95% CI) | 0.89 (0.60-1.32)^a^ | 1.05 (0.67-1.63) |
| EVT-LA vs. BMT  OR (95% CI) | 1.12 (0.70-1.81)^a^ | 1.32 (0.77-2.30) |
| EVT-CS vs. BMT  OR (95% CI) | 0.57 (0.33-0.99)^a^ | 0.82 (0.46-1.49) |
| **Sensitivity analysis 2** |  |  |
| EVT-GA vs. BMT  OR (95% CI) | 0.89 (0.61-1.31)^a^ | 1.04 (0.68-1.59) |
| EVT-LA vs. BMT  OR (95% CI) | 1.21 (0.76-1.93)^a^ | 1.41 (0.84-2.43) |
| EVT-CS vs. BMT  OR (95% CI) | 0.58 (0.34-0.98)^a^ | 0.80 (0.45-1.42) |
| **Sensitivity analysis 3** |  |  |
| EVT-non-GA vs. BMT  OR (95% CI) | 0.87 (0.58-1.29)^a^ | 1.09 (0.71-1.70) |

Sensitivity analysis 1: Patients who crossed over from BMT to EVT were included in an EVT group based on the anesthesia they received, and those who did not receive any passes were included in the BMT group irrespective of whether they had already received some sedation. BMT (n=299), EVT-GA (n=109), EVT-LA (n=65), EVT-CS (n=52).

Sensitivity analysis 2: Patients who crossed over to BMT were allocated to the BMT group only if they did not receive any anesthesia, whereas those who received GA, CS, or LA were assigned to the respective EVT groups, irrespective of whether EVT was performed. BMT (n=269), EVT-GA (n=126), EVT-LA (n=73), EVT-CS (n=57).

Sensitivity analysis 3: EVT-LA and EVT-CS combined into the EVT-non-GA group and compared to BMT alone. EVT-non-GA (n=116), BMT alone (267).

^a^Common OR. BMT, best medical treatment; EVT, endovascular treatment; GA, general anesthesia; LA, local anesthesia; CS, conscious sedation; OR, odds ratio; CI, confidence interval; mRS, modified Rankin Scale.

**Table S2.** Unadjusted sensitivity analyses on outcome comparison between different anesthesia modalities during endovascular treatment

|  | **mRS at 90 d** | **mRS 0-2 at 90 d** |
| --- | --- | --- |
| **Sensitivity analysis 4** |  |  |
| EVT-non-GA vs. EVT-GA  OR (95% CI) | 1.08 (0.66-1.76)^a^ | 1.06 (0.62-1.79) |
| **Sensitivity analysis 5** |  |  |
| EVT-any sedatives vs. EVT-LA  OR (95% CI) | 0.65 (0.38-1.1)^a^ | 0.70 (0.38-1.26) |

Sensitivity analysis 4: EVT-LA and EVT-CS combined into an EVT-non-GA group and compared to EVT-GA. EVT-non-GA (n=116), EVT-GA (n=108).

Sensitivity analysis 5: EVT-CS and EVT-GA combined into an EVT-any sedatives group and compared to EVT-LA. EVT-CS + EVT-GA (n=160), EVT-LA (n=64)

^a^Common OR. BMT, best medical treatment; EVT, endovascular treatment; GA, general anesthesia; LA, local anesthesia; CS, conscious sedation; OR, odds ratio; CI, confidence interval; mRS, modified Rankin Scale.
